# Supplementary material for: Hemodynamic and electromechanical effects of paraquat in rat heart
Source: PLoS One. 2021 Apr 1;16(4):e0234591. doi: 10.1371/journal.pone.0234591 (PMC8016255; doi:10.1371/journal.pone.0234591)
Supplement: S1 Fig — (DOCX) [file pone.0234591.s001.docx]

Supplementary Fig S1. Representative recordings of arterial pressure, LV pressure (LVP), first derivative of LV pressure (LV d*P*/d*t*), and ECG from an anesthetized rat at baseline and at various times after normal saline treatment (1 mL/kg, i.v.). QTc value in each panel denotes rate-corrected QT interval derived using normalized Bazett’s formula QTc = QT/(RR/*f*)^1/2^, where *f* = 180 ms.
