# Supplementary material for: Hemodynamic and electromechanical effects of paraquat in rat heart
Source: PLoS One. 2021 Apr 1;16(4):e0234591. doi: 10.1371/journal.pone.0234591 (PMC8016255; doi:10.1371/journal.pone.0234591)
Supplement: S2 Fig — (DOCX) [file pone.0234591.s002.docx]

Supplementary Fig S2. Effects of saline vehicle (0.03% v/v) on LV pressure (LVP) in Langendorff-perfused isolated rat hearts paced at 300 beats/min. (A) A representative tracing showing that saline administration failed to alter LVP in a perfused heart. (B) Representative tracings of LVP (upper) and LV d*P*/d*t* (lower) on an expanded time scale recorded before and after application of saline. (C) Mean values of LV developed pressure, +d*P*/d*t*_max_, and –d*P*/d*t*_max_ before and 30 and 60 min after application of saline. All data are expressed as mean ± SD (*n* = 8).
