# Supplementary material for: Hemodynamic and electromechanical effects of paraquat in rat heart
Source: PLoS One. 2021 Apr 1;16(4):e0234591. doi: 10.1371/journal.pone.0234591 (PMC8016255; doi:10.1371/journal.pone.0234591)
Supplement: S3 Fig — (DOCX) [file pone.0234591.s003.docx]

**A** **B**

**C** **D**

**Supplementary Fig S3.** Effects of paraquat on kinetic parameters of cell shortening and intracellular Ca^2+^ transients (represented by fura-2 fluorescence ratio *F*_340_/*F*_380_) in rat ventricular myocytes. (A, B) Effects of PQ on time-to-peak of cell shortening (A) and time to 50% of cell relengthening (B). (C, D) Effects of PQ on time-to-peak (C) and decay time constant (D) of Ca^2+^ transients. All data are expressed as mean ± SD (*n* = 11).
