# Supplementary material for: Hemodynamic and electromechanical effects of paraquat in rat heart
Source: PLoS One. 2021 Apr 1;16(4):e0234591. doi: 10.1371/journal.pone.0234591 (PMC8016255; doi:10.1371/journal.pone.0234591)
Supplement: S4 Fig — (DOCX) [file pone.0234591.s004.docx]

**Supplementary Fig S4.** Effects of saline on Ca^2+^ transients (represented by fura-2 fluorescence ratio *F*_340_/*F*_380_) and cell shortening in rat ventricular myocytes paced at 1 Hz. (A) Continuous recordings of Ca^2+^ transients (upper) and cell shortening (lower) showing the effects of cumulative application of 0.1, 0.3, and 0.6 % (v/v) saline in a chamber with 1 mL buffer solution. (B) Recordings on an expanded time scale taken at the time indicated by the corresponding letters in A. (C) The mean data of the amplitude and kinetic parameters of Ca^2+^ transient (upper) and cell shortening (lower) before and after application of saline. Data are expressed as mean ± SD (*n* = 12). Cell shortening was normalized to resting cell length.
