# Supplementary material for: Hemodynamic and electromechanical effects of paraquat in rat heart
Source: PLoS One. 2021 Apr 1;16(4):e0234591. doi: 10.1371/journal.pone.0234591 (PMC8016255; doi:10.1371/journal.pone.0234591)
Supplement: S1 Table — (DOCX) [file pone.0234591.s005.docx]

**Supplementary Table 1** Baseline hemodynamic and electrocardiographic parameters in anesthetized rats receiving the vehicle or PQ at a dose of 100 or 180 mg/kg

| Variables | Saline | (*n*=10) | 100 mg/kg (*n*=10) | | | 180 mg/kg (*n*=10) | | | *p* value |
| --- | --- | --- | --- | --- | --- | --- | --- | --- | --- |
| Body Weight (g) | 281.5 | ± 42.6 | 268.5 | ± | 31.8 | 290.5 | ± | 22.7 | 0.35 |
| HR (beats/min) | 345 | ± 41 | 322 | ± | 45 | 334 | ± | 28 | 0.42 |
| SBP (mmHg) | 104.2 | ± 22.7 | 117.0 | ± | 9.2 | 109.5 | ± | 15.3 | 0.24 |
| DBP (mmHg) | 48.0 | ± 9.8 | 48.7 | ± | 8.5 | 44.9 | ± | 11.0 | 0.66 |
| MAP (mmHg) | 68.3 | ± 11.7 | 71.8 | ± | 7.5 | 68.8 | ± | 10.1 | 0.70 |
| LVESP (mmHg) | 105.8 | ± 13.6 | 104.1 | ± | 8.5 | 110.8 | ± | 17.4 | 0.53 |
| LVEDP (mmHg) | 4.6 | ± 3.0 | 2.1 | ± | 3.1 | 1.2 | ± | 4.6 | 0.12 |
| +d*P*/d*t*_max_ (mmHg/s) | 9537 | ± 1987 | 9357 | ± | 1228 | 7827 | ± | 3132 | 0.20 |
| –d*P*/d*t*_max_ (mmHg/s) | –4980 | ± 1064 | –4701 | ± | 998 | –4486 | ± | 1768 | 0.71 |
| P wave (ms) | 28.7 | ± 5.9 | 24.6 | ± | 3.3 | 29.3 | ± | 7.8 | 0.18 |
| PR (ms) | 60.4 | ± 8.4 | 65.8 | ± | 5.5 | 60.1 | ± | 6.9 | 0.14 |
| QRS (ms) | 14.5 | ±1.1 | 14.2 | ± | 1.4 | 14.1 | ± | 1.5 | 0.86 |
| QT (ms) | 65.7 | ± 12.7 | 72.8 | ± | 16.3 | 75.3 | ± | 9.6 | 0.26 |
| QTc (ms) | 66.2 | ± 15.8 | 74.3 | ± | 15.8 | 79.6 | ± | 7.4 | 0.44 |

Data are expressed as mean ± SD. HR, heart rate; SBP, systolic blood pressure; DBP, diastolic blood pressure; MAP, mean arterial blood pressure; LVESP, left ventricular end-systolic pressure; LVEDP, left ventricular end-diastolic pressure; +d*P*/d*t*_max_ and –d*P*/d*t*_max_, maximal rate of rising and fall of LV pressure, respectively. QTc, rate-corrected QT interval derived using normalized Bazett’s formula QTc = QT/(RR/*f*)^1/2^, where *f* = 180 ms.
